# Biogeography and evolution of a widespread Central American lizard species complex: *Norops humilis*, (Squamata: Dactyloidae)

**DOI:** 10.1186/s12862-015-0391-4

**Published:** 2015-07-19

**Authors:** John G. Phillips, Jennifer Deitloff, Craig Guyer, Sara Huetteman, Kirsten E. Nicholson

**Affiliations:** 1 Department of Biology, Central Michigan University, Mt. Pleasant, MI 48859 USA; 2 Present address: Department of Biological Sciences, University of Tulsa, Tulsa, OK 74104 USA; 3 Department of Biological Sciences, Auburn University, Auburn, AL 36849 USA; 4 Present address: Department of Biological Sciences, Lock Haven University, Lock Haven, PA 17745 USA

**Keywords:** Ancestral range estimation, Anoles, Dispersal, Internal transcribed spacer (ITS), Mesoamerica, mtDNA, Neotropical diversity, Reptilia, Spatial Analysis of Molecular Variance (SAMOVA), Squamata

## Abstract

**Background:**

Caribbean anole lizards (Dactyloidae) have frequently been used as models to study questions regarding biogeography and adaptive radiations, but the evolutionary history of Central American anoles (particularly those of the genus *Norops)* has not been well studied. Previous work has hypothesized a north-to-south dispersal pattern of Central American *Norops*, but no studies have examined dispersal within any *Norops *lineages. Here we test two major hypotheses for the dispersal of the *N. humilis/quaggulus* complex (defined herein, forming a subset within Savage and Guyer’s *N. humilis* group).

**Results:**

Specimens of the *N. humilis* group were collected in Central America, from eastern Mexico to the Canal Zone of Panama. Major nodes were dated for comparison to the geologic history of Central America, and ancestral ranges were estimated for the *N. humilis/quaggulus* complex to test hypothesized dispersal patterns. These lineages displayed a northward dispersal pattern. We also demonstrate that the *N. humilis/quaggulus* complex consists of a series of highly differentiated mitochondrial lineages, with more conserved nuclear evolution. The paraphyly of the *N. humilis* species group is confirmed. A spatial analysis of molecular variance suggests that current populations are genetically distinct from one another, with limited mitochondrial gene flow occurring among sites.

**Conclusions:**

The observed south-to-north colonization route within the *Norops humilis/quaggulus* complex represents the first evidence of a *Norops* lineage colonizing in a south-to-north pattern, (opposite to the previously held hypothesis for mainland *Norops*). One previously described taxon (*N. quaggulus*) was nested within *N. humilis*, demonstrating the paraphyly of this species; while our analyses also reject the monophyly of the *Norops humilis* species group (*sensu* Savage and Guyer), with *N. tropidonotus, N. uniformis,* and *N. marsupialis* being distantly related to/highly divergent from the *N. humilis/quaggulus* complex. Our work sheds light on mainland anole biogeography and past dispersal events, providing a pattern to test against other groups of mainland anoles.

**Electronic supplementary material:**

The online version of this article (doi:10.1186/s12862-015-0391-4) contains supplementary material, which is available to authorized users.

## Background

Central America is an important region for understanding historical biogeography and intercontinental dispersal in the Western Hemisphere. Mesoamerica has served as a pathway of dispersal for many taxa, some of which originated in North America and dispersed south (e.g. Pitvipers [1], but see [2]; Bolitoglossine salamanders [3]) and others that moved north following a southern origin (e.g. hylid frogs [4]; toads [5]). Lower Central America (LCA) displays extraordinary high levels of species diversity in many taxonomic groups including insects [6], fish [7, 8] and herpetofauna [9, 10]. Many phylogeographic studies in LCA have been conducted on a diverse assemblage of organisms, including trees [11, 12], mammals [13–15], fish [16, 17], and amphibians [3, 18, 19]. In reptiles, there have been some work on squamate biogeography [1, 20–24], but the majority of these studies have centered on snakes, with no published multi-locus phylogeographic studies on lizards in Central America (Hasbún et al. [25] uses a single mtDNA marker to investigate biodiversity in a species of *Ctenosaura*). Given the rich history of scientific work on lizards in the Caribbean, especially anoles (family Dactyloidae [26–29]), the lack of biogeographic work on mainland lizards is somewhat surprising.

**Fig. 1:**
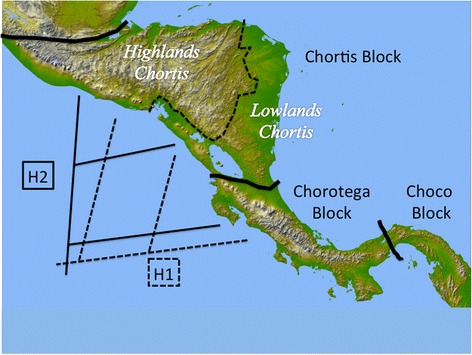
Representation of two alternative dispersal hypotheses for the *N. humilis/quaggulus* complex investigated in this study. Solid black lines demark the rough geologic boundaries of major tectonic blocks as they correspond to present-day Central America. The dotted line marks the subdivision of the Chortis block into highland and lowland regions. The phylogenetic representation of the first dispersal hypothesis (H1) is indicated by the dotted lines; the alternative (H2) is shown with solid lines

Anoles, present throughout Central America [10, 30], are an ideal group to remedy this deficiency. Several species groups in the genus *Norops* are widespread, and present excellent opportunities to study the colonization of Mesoamerica. Among these taxa, we selected the *Norops humilis* species group as a model for refining biogeographic hypotheses for Central American lizards in general, and Central American anoles in particular. The *N. humilis* species group (described below) ranges from Mexico to Panama, although the timing of diversification within this group has not been determined. Our first objective was to estimate the date of origin for the group, which may in part provide support for one of two hypotheses. Hypothesis 1 (H1; see Fig. 1) predicts a north-to-south dispersal pattern, while Hypothesis 2 (H2; see Fig. 1) predicts a south-to-north dispersal pattern. Our second objective was to test biogeographic patterns within this group, as interpreted from the above hypotheses. The *N. humilis* species group [31] is an ideal group for examining these hypothesized patterns as well as their timing. As originally defined, the group includes *N. compressicauda*, *N. humilis*, *N. notopholis*, *N. tropidonotus*, and *N. uniformis*. To these we add the newly described *N. wampuensis* [32] and *N. quaggulus* [33], plus *N. marsupialis*, a former subspecies of *N. humilis* that has recently been treated as a species [34]. Several studies suggest the *N. humilis* species group may be paraphyletic [35–37]. Therefore, our third objective was to confirm the polyphyly of the *N. humilis* species group. We also examined the *N. humilis/quaggulus* complex, which we define to include *N. humilis* plus *N. quaggulus*, a sister species described based on putatively unique hemipenial morphology and evidence of reciprocal monophyly based on mitochondrial genes [33]. This complex also has a wide distribution (Panama to Honduras). Our analyses provide the opportunity to further evaluate the relationship between *N. humilis* and *N. quaggulus*, as well as to test the specific status of *N. marsupialis*.

## Methods

**Fig. 2:**
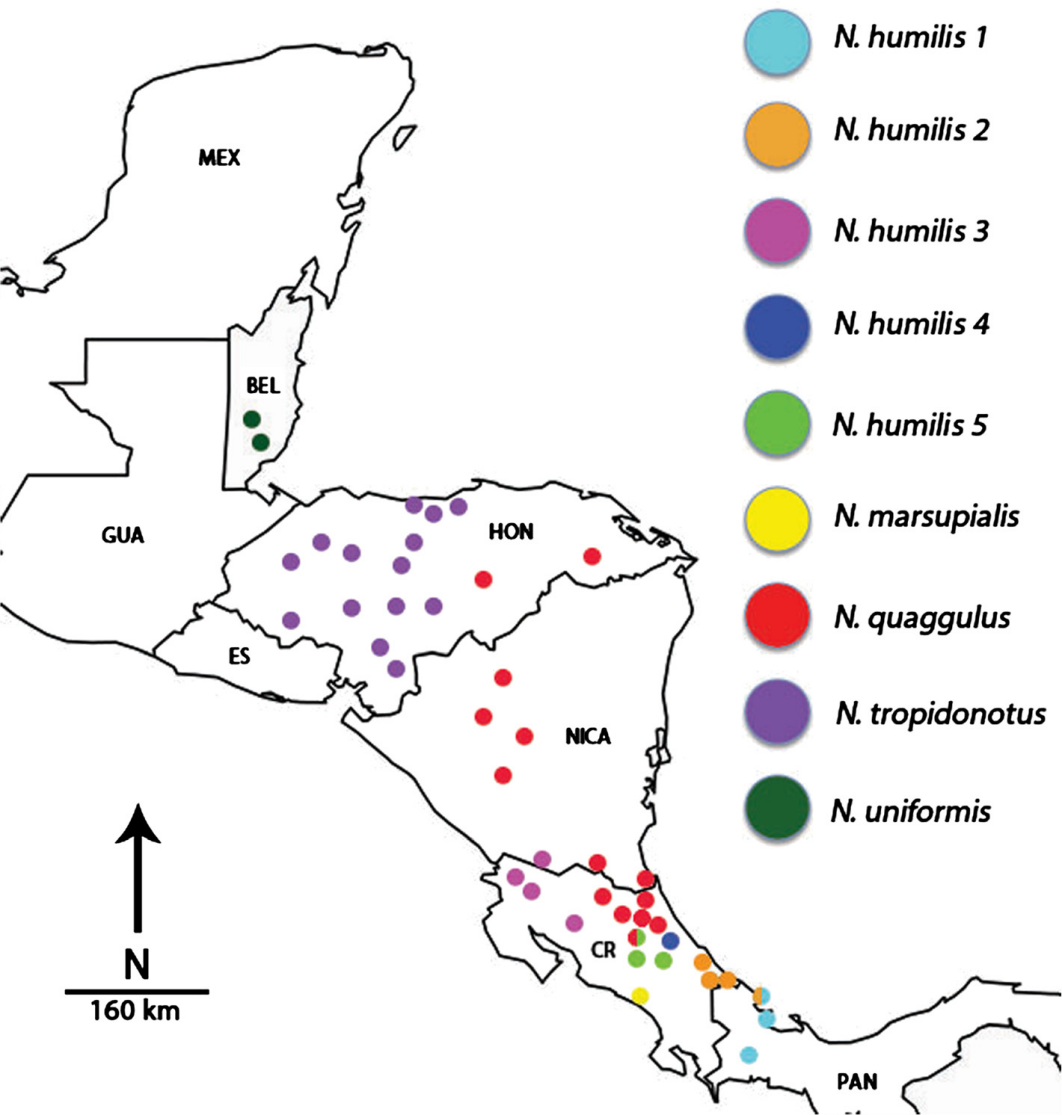
Geographic distribution of *Norops humilis* species group lineages used in this study. This map of the *Norops humilis* species group denotes the six main lineages in the *N. humilis/quaggulus* complex as hypothesized by the phylogenetic reconstructions from the Maximum Likelihood and Bayesian analyses. Note that two localities contain multiple lineages of *N. humilis*. An additional site for *N. tropidonotus* from Veracruz, Mexico, is not included in this map. Abbreviations: PAN = Panama, CR = Costa Rica, NICA = Nicaragua, HON = Honduras, ES = El Salvador, GUA = Guatemala, BEL = Belize and MEX = Mexico

Samples from several of the species in the *Norops humilis* species group (*N. humilis/quaggulus*, *N. marsupialis*, *N. tropidonotus* and *N. uniformis*) were collected throughout Central America or acquired from tissue loans (Fig. 2, Additional file 1: Appendix S1). For the *N. humilis/quaggulus* complex, 147 specimens were sampled throughout Honduras, Nicaragua, Costa Rica, and Panama. DNA was extracted from liver or muscle tissues using Qiagen DNeasy kits (Qiagen, USA). PCR was conducted following protocol and using lizard-specific primers from Macey et al. [38] for mtDNA and Nicholson [35] for nucDNA. Purified PCR reactions were sent to Michigan State University’s Research and Technology Support Facility for sequencing of the following gene regions: NADH-ubiquinone oxidoreductase chain 2 (ND2), tRNA^Trp^, tRNA^Ala^, tRNA^Asn^, tRNA^Cys^, tRNA^Tyr^, origin of light strand replication, and partial CO1 from mitochondrial DNA. A subset of these samples representing each major lineage detected by the mitochondrial analyses was selected for nuclear DNA analysis using a nuclear internal transcribed spacer unit (ITS-1). A nuclear marker was included because many studies have shown the limitations of mtDNA to reflect levels of gene flow or the extent of reproductive isolation among populations (e.g. [39, 40]; see [41] for a review). Analyses based solely on mtDNA can also provide results that are in conflict with the nuclear genome [42, 43].

Within the *N. humilis* species group, a total of 1451 aligned bp of mitochondrial data were collected for 192 individuals and 1522 aligned bp of the nuclear gene region ITS-1 was collected for 48 individuals. All newly acquired data were combined with published sequences for 65 additional *Norops* species (Additional file 2: Appendix S2) in order to investigate the monophyly of the *N. humilis* species group and of the *N. humilis/quaggulus* complex. Sequences were edited using Sequencher 4.9 (GeneCodes Corp., Ann Arbor, MI, USA) and aligned initially using MUSCLE in MEGA 5.2.2 [44], then adjusted manually.

The relatively continuous geographic distribution of the *N. humilis/quaggulus* complex combined with a lack of distinct phenotypic differences made *a priori* separation of sampling localities into populations somewhat arbitrary. In order to describe the genetic structure and identify the best maximally differentiated number of populations within the *N. humilis/quaggulus* complex, a spatial analysis of molecular variance (SAMOVA 1.0) was used to group 15 localities selected in this study (each with *n* ≥ 4 specimens; Additional file 3: Appendix S3) into a number of user-defined clusters (*K*). For each cluster, the proportion of total genetic variance (high *F*_*CT*_ index) due to differences between populations [45] was estimated and evaluated to select the optimal number of genetic groups. A simulated annealing process for each cluster (*K* = 2 to 14) was repeated 1023 times for each of 100 sets of initial conditions to ensure that the final population groups were not affected by the initial configuration. Significance of the *F*_*SC*_ index was used to obtain the suggested number of genetic groupings for the localities selected [46]. This analysis was based on sequences for the mitochondrial region only, since ITS-1 data were only obtained for a limited number of specimens, and were not sufficient for population genetic analysis.

Phylogenetic estimations were conducted under Bayesian analytical methods. PARTITIONFINDER [47] was used to select models of evolution as well as to examine the suitability of partitioning each dataset (mitochondrial, nuclear, and concatenated). In all cases each gene (including tRNAs) was entered as a potential partition. Protein coding genes were further partitioned by codon position for the PartitionFinder analysis. Branch lengths were unlinked, all models of evolution available in MRBAYES 3.2.2 [48] were tested, and a BIC information criterion and greedy algorithm were used. The PARTITIONFINDER analysis recommended a HKY + I + Γ model of evolution for the mtDNA segment, and GTR + Γ for the nuclear data with no partitioning recommended within either region. For the combined dataset, two partitions were recommended (mt and nuc) with GTR + I + Γ as the selected model of evolution for both partitions.

Bayesian analyses for each dataset (as above) were conducted using MRBAYES and BEAST 1.7.5 [49]. The phylogenetic hypotheses using MRBAYES 3.2.2 was developed using 20 million generations, sampling every 1000 generations for two independent runs using four Markov chains with node support evaluated via posterior probabilities (BAPP). We evaluated stationarity of variables by examining our output via TRACER v1.5 [50]. The first 20 % of trees were discarded as burnin and a majority rule consensus tree was generated to summarize the post-burnin results. With the phylogeny constructed using BEAST, we estimated divergence dates, employing a lognormal relaxed clock and a calibration rate of 0.65 % per million years for mtDNA [38] with a Yule Process speciation prior. This rate has been used for our selected mitochondrial gene region for reptile and amphibian groups [51, 52] including anoles (e.g. [53, 54]; mean of a prior distribution, SD = 0.0025 for ucld.mean parameter). The calibration rate was only applied to the mtDNA analysis, as there are no calibration rates available for the nuclear ITS-1 gene. In addition to Bayesian analyses, ML analyses were conducted on each dataset using MEGA 5.2.2 with node support evaluated via bootstrap analyses (MLBS) based on 2000 replications. In addition to the constructed topologies, pairwise genetic distance (uncorrected-p) was estimated between all individuals included in the phylogenetic analyses using both the mitochondrial and concatenated datasets. These distances were compared among all major lineages (as indicated by phylogenetic analysis) of *N. humilis* species group members, as well as within each species or lineage using MEGA 5.2.2.

Phylogeographic hypotheses were tested using likelihood-based inference in LAGRANGE [55, 56]. The analysis was conducted using the phylogeny constructed with BEAST for mtDNA only, as divergence dating was not available for the nuclear (ITS) tree. Dispersal events between regions were examined and ancestral ranges were estimated within the *N. humilis/quaggulus* complex to evaluate the support for each of the hypotheses presented above (Fig. 1). The geographic regions coded were (1) the Caribbean versant of the Chorotega Block, (2) the Pacific versant of the Chorotega Block, (3) the lowlands of the Chortis Block and (4) Highlands of the Chortis Block. These areas represent the entire range of the *N. humilis/quaggulus* complex and were selected based on major geographic barriers. For the analysis, dispersal was constrained to be possible only between adjacent regions because the anoles studied herein are both small and ground dwelling, which would make rapid dispersal to very distant and non-adjacent regions unlikely. If the complex originated in the northern part of the range (i.e. Honduras or northern Nicaragua) with younger clades in the south, it would indicate a north-to-south dispersal, while a southern origin would support a south-to-north colonization route.

## Results

**Fig. 3:**
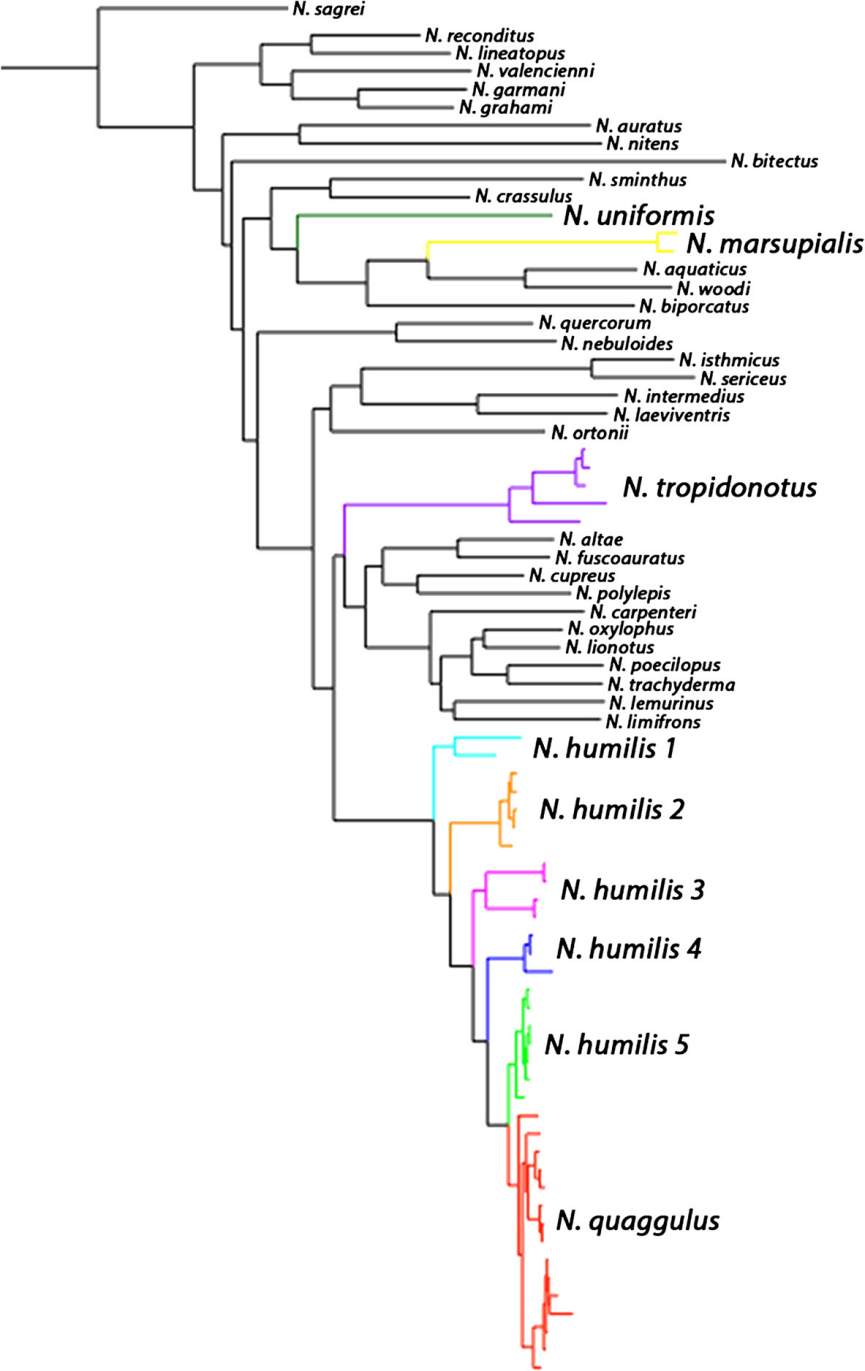
Bayesian phylogenetic hypothesis from both mitochondrial and nuclear genes combined. Bayesian posterior probabilities are located at all major nodes

**Fig. 4:**
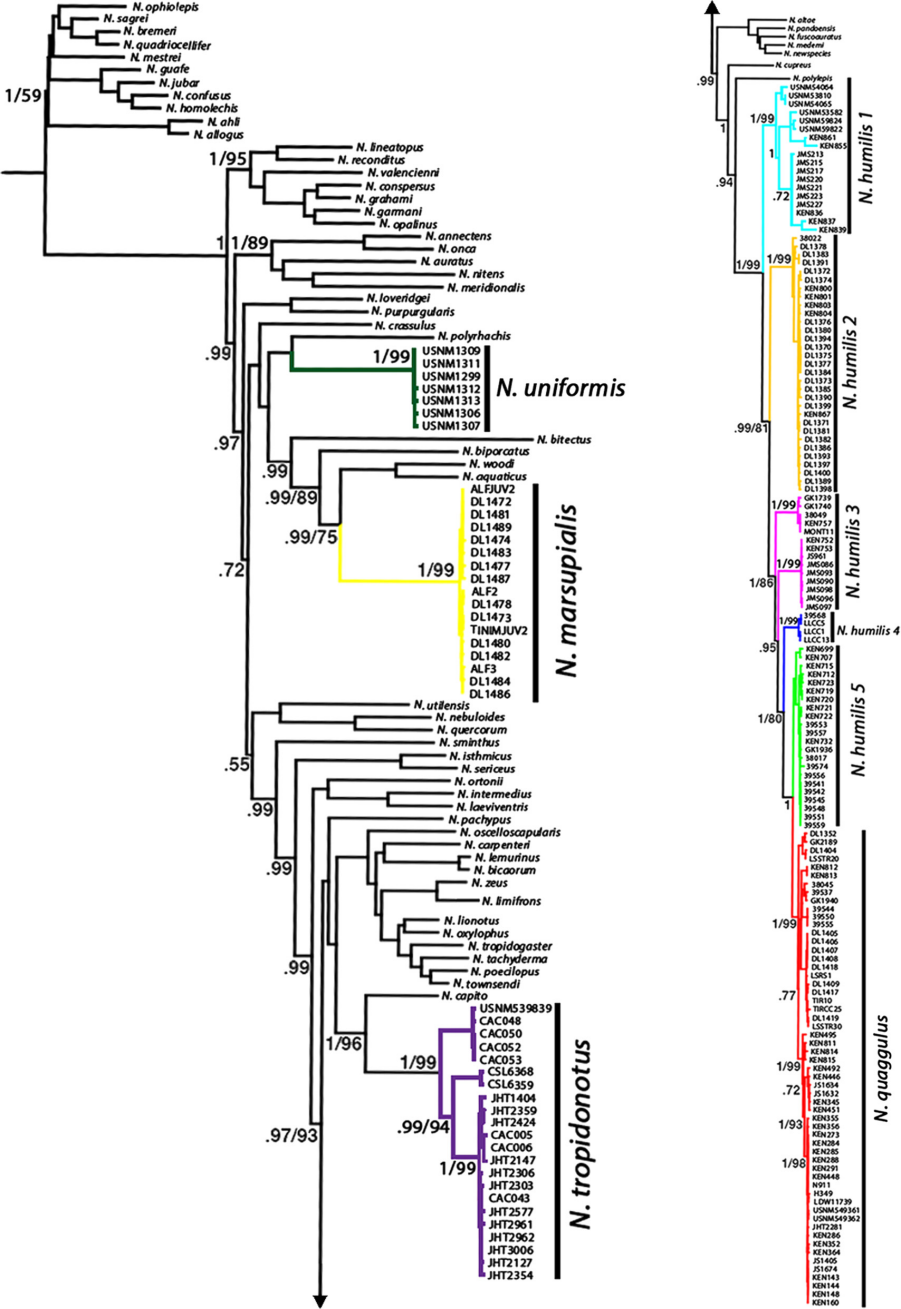
Bayesian reconstruction of *Norops* using the mitochondrial genes. Genes used were ND2, tRNA^Trp^, tRNA^Ala^, tRNA^Asn^, tRNA^Cys^, tRNA^Try^, origin of light strand replication, and partial CO1, including multiple samples of *N. humilis* species group. The numbers at nodes are posterior probabilities followed by bootstrap values for nodes that were congruent between the analyses (i.e. posterior/bootstrap)

**Fig. 5:**
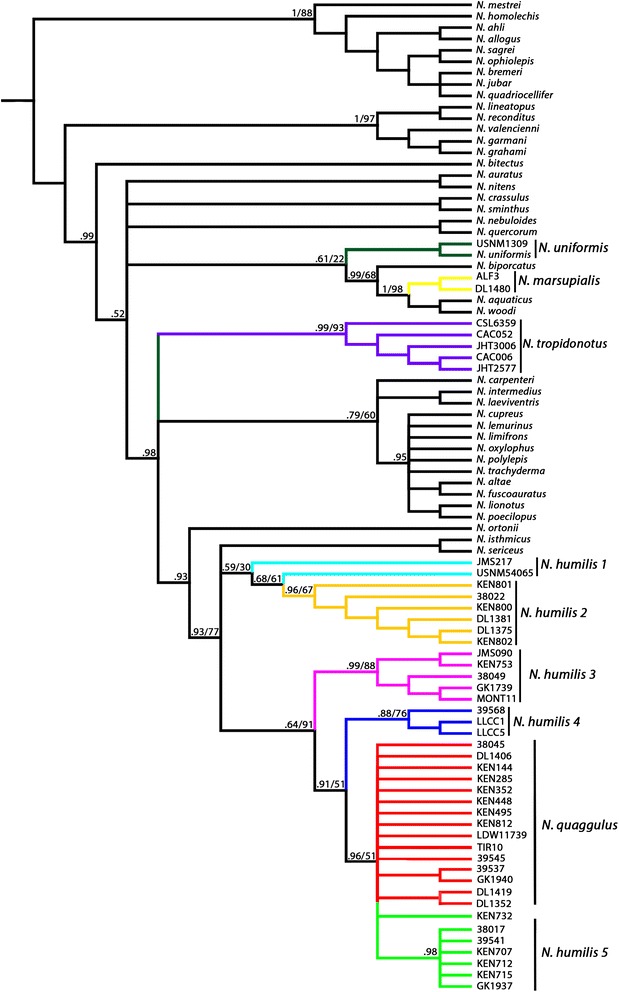
Bayesian reconstruction of *Norops* using the nuclear gene ITS-1. This tree includes multiple samples of *N. humilis* species group members. The numbers at nodes are posterior probabilities followed by bootstrap values for nodes that were congruent between the analyses (i.e. posterior/bootstrap)

**Fig. 6:**
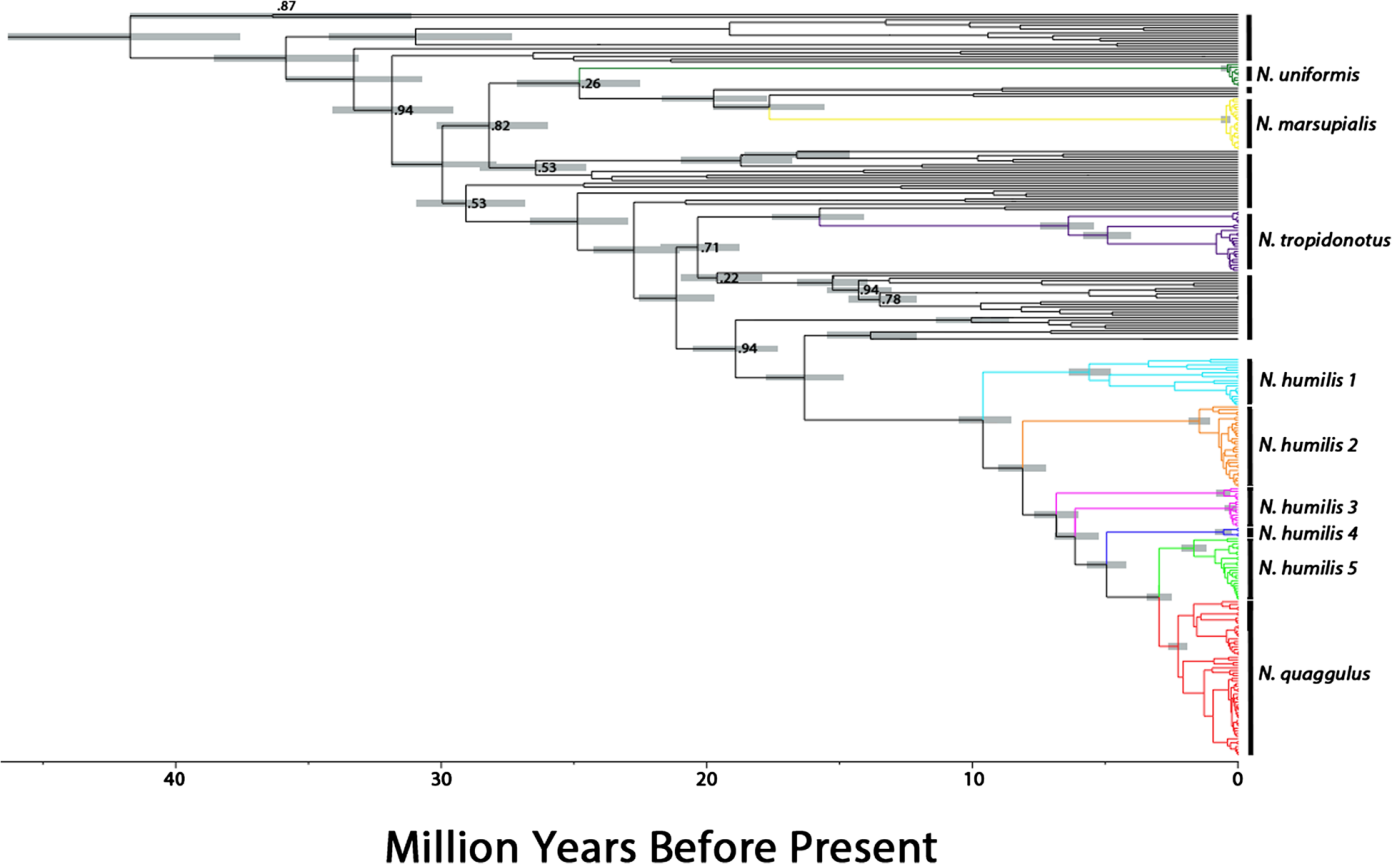
Divergence dating of the *N. humilis* group in relation to other *Norops*. Tree was created in BEAST 1.7.5 using a calibration rate from Macey et al. [38]. Nodes with <0.95 posterior probabilities have their values listed on the tree. Where present, all grey bars on the nodes correspond to the 95 % confidence interval of the date

All phylogenetic hypotheses demonstrate the paraphyly of the *N. humilis* species complex (Figs. 3, 4, 5 and 6). Each phylogenetic analysis is discussed in detail below in regard to the *N. humilis/quaggulus* complex. Six main lineages were present when using the concatenated dataset (mt + nucDNA) for both Bayesian (Fig. 3) and ML (not shown) phylogenetic analyses. One lineage (lineage 6) corresponded to samples currently classified as *N. quaggulus:* a clade nested within the rest of the *N. humilis/quaggulus* complex, rendering the current species designation of *N. humilis* paraphyletic.

The trees from our mitochondrial analyses were similar in topology to those from the combined data with the only difference being a lineage of the *N. humilis/quaggulus* complex restricted to the Pacific versant of Costa Rica and Nicaragua (*N. humilis* lineage 3, Figs. 2, 3, 4, 5 and 6). In the Bayesian analysis of mtDNA, this lineage formed two separate clades: one was comprised of samples from Monteverde, Costa Rica and the other was comprised of all additional nearby samples from Guanacaste, Costa Rica plus specimens from southern Nicaragua (Fig. 4). These samples formed a single lineage for the concatenated dataset using both Bayesian (Fig. 3) and ML as well as for the mtDNA dataset using ML. We also constructed a phylogenetic tree on the mtDNA dataset with BEAST. Results of the BEAST analysis provided a topology in agreement with the tree constructed using MRBAYES for all major lineages (Fig. 6). When considering 0.65 % per lineage per million years for the mtDNA data, the origin of the *N. humilis/quaggulus* complex was estimated to be 17.2 Myr BP (95 % CI = 14.2-20.6 Myr BP). The stem age for each of the distinct mitochondrial lineages of the *N. humilis/quaggulus* complex were estimated to have originated between 10.3 and 3.2 Myr BP (Fig. 6).

When using only the nuclear ITS-1 dataset, lineages were less divergent than either mitochondrial dataset described above. The Bayesian analyses differed slightly from the mtDNA and combined analyses by presenting only four main clades of the *N. humilis/quaggulus* complex (Fig. 5). Primary differences included the presence of a conjoined Panamanian/eastern Costa Rican clade (lineages 1 and 2), a single Pacific Costa Rican clade (monophyletic lineage 3), one clade (lineage 4) of a single Caribbean Costa Rican locality (la Lola, Limon Province) and the remaining Caribbean Costa Rican samples (lineages 5 and 6) grouping together in an unresolved clade. The ML analysis of ITS-1 reduced the *N. humilis/quaggulus* complex to three main clades: Eastern Costa Rica/Panama (lineages 1 and 2), Pacific Costa Rica plus one Caribbean site (lineages 3 and 4), and the remaining Caribbean Costa Rican samples (lineages 5 and 6; figure not shown). However, the clades in the ML analysis lack significant support, possibly due to the conservative nature of ITS-1, which failed to yield significant variation among closely related lineages.

When we calculated pairwise genetic distances using the concatenated dataset, genetic distance (uncorrected p) was very high between the *N. humilis/quaggulus* complex and other members of the *N. humilis* species group: 18-20 % from *N. tropidonotus,* 20–25 % from *N. uniformis,* and 21–26 % from *N. marsupialis*. In addition, within the *N. humilis/quaggulus* complex, genetic distances (uncorrected p, Table 1) ranged up to 10 % among all *N. humilis/quaggulus* complex lineages. Distances within lineage 6, shown as *N. quaggulus*, ranged up to 3 %, and were up to 9 % within each of the other lineages, shown as *N. humilis* 1–5 (>2 % for all clades except *N. humilis* lineage 3).

**Table 1:** Genetic distance among members of the Norops humilis species group (sensu Savage and Guyer [31])

Distances for all N. quaggulus samples are pooled; samples of N. humilis are separated out by clade as defined by the combined Bayesian analysis (Fig. 3). Distances for mtDNA data are italicized, while distances for the combined nuclear and mtDNA data are in bold

SAMOVA was used to designate the optimal number of distinct genetic clusters within the *N. humilis/quaggulus* complex. Grouping of the 15 sample localities into 12 clusters by SAMOVA yielded an *F*_*SC*_ value (0.03) that approached zero (Additional file 3: Appendix S3). A lack of shared haplotypes among the localities used in the analysis indicated limited gene flow, at least for the genes in question. *K* = 12 was chosen as the most likely number of clusters, because it maximized the variation among clusters (*F*_*CT*_ = 0.86) while minimizing the variation among localities within clusters (*F*_*SC*_). While *F*_*CT*_ increased for *K* = 13 and *K* = 14, the difference was slight (*F*_*CT*_ = 0.86 and 0.87 respectively). Rodríguez-Robles et al. [46] stated that *F*_*CT*_ should peak where *F*_*SC*_ = 0, so we use *F*_*SC*_ as the determining factor in our analysis, given the low magnitude of increases in *F*_*CT*_ created by additional partitioning. The variance among clusters (85.5 %) accounted for the majority of genetic diversity within the *N. humilis/quaggulus* complex (*F*_*CT*_ = 0.86), 0.4 % by the variation among localities within these clusters (*F*_*SC*_ = 0.03), and 14.1 % by the variation among each of the localities (*F*_*ST*_ = 0.86). The 12 populations identified in the *N. humilis/quaggulus* complex by the SAMOVA analysis was double the number of major lineages we designate in our phylogenetic analyses (*n* = 6), because the population analysis identified additional substructure that was not recovered in our phylogenetic hypothesis.

**Fig. 7:**
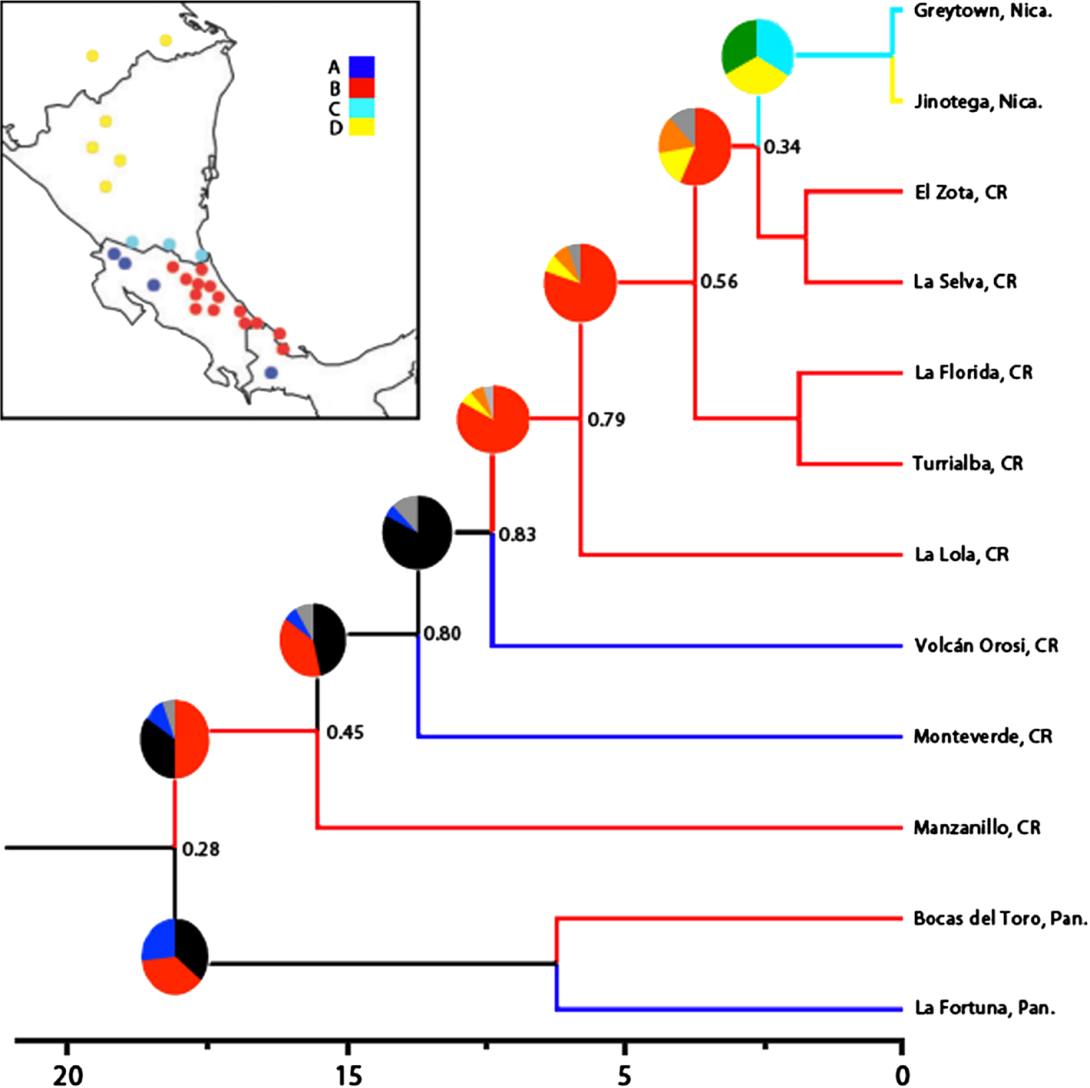
Ancestral range estimation of the *Norops humilis/quaggulus* complex created using LAGRANGE. The probabilities for the present scenario are indicated at each node unless the scenario has a probability of 1, with all probabilities reflected on each internal branch. Probabilities correspond to the branches stemming from a node, rather than the node itself, they are not reflective of the confidence in the topology. Geographic regions are coded as follows: Pacific versant of the Chorotega Block (A), Caribbean versant of the Chorotega Block (B), lowlands Chortis Block (C) and highlands Chortis Block (D). Branches indicating simultaneous occupation of regions are black (AB), orange (BC), yellow (BD), green (CD), or gray (three or more regions). PLEASE NOTE the colors on this figure pertain to geographical regions, whereas in all other figures correspond to lineages. The tree used for this analysis was created in BEAST v1.7.5 and only contains mitochondrial data since divergence dating was not possible for ITS-1. All nodes included in this tree are strongly supported (posterior probabilities > 0.95)

The LAGRANGE analysis using the tree recovered from BEAST yielded a log likelihood of –lnL = 22.4 at the root node with dispersal and extinction probabilities of 0.01 and 7.1e-10 respectively. The ancestral range estimation indicates a south-to-north distribution for the *N. humilis/quaggulus* complex (Fig. 7). The Panamanian lineage (*N. humilis* 1) is sister to the rest of the *N. humilis/quaggulus* complex. A clade containing all Honduras and Caribbean Nicaragua specimens was younger than any clades restricted to Costa Rican or Panamanian samples in both ML and Bayesian analyses. Coupled with our ancestral range estimation, the topology suggests that the *N. humilis/quaggulus* complex originated in the south before dispersing northwards.

## Discussion and conclusions

To address our first objective, we estimated the age of the *Norops humilis/quaggulus* species complex and evaluated two potential dispersal patterns for this group. The BEAST analysis yielded a mean crown group age of 17.2 Myr BP (range = 14.2–20.6) for the clade. We report this date cautiously because it was calculated from mitochondrial data only. To evaluate the second objective, we used the ancestral range estimation to evaluate dispersal patterns. Using the LAGRANGE output, we infer that the common ancestor of the *N. humilis/quaggulus* complex originated in Panama before dispersing west to Costa Rica, and then north into Nicaragua and Honduras. The south-to-north pattern and origin in Panama suggests isolation of the ancestor of the *N. humilis/quaggulus* complex in the Talamancan region of extreme LCA. The divergence between Pacific and Caribbean *N. humilis* in northern Costa Rica (Chorotega Block) was estimated to be 6.6 Myr BP (range = 4.12–6.94), which corresponds to the estimate of 5.4 Myr BP for the rise of the lower Central American highlands [57]. This uplift represents a potential vicariant event responsible for the separation of the Pacific (lineage 3) and Caribbean lineages (*N. humilis* lineages 4 and 5 + *N. quaggulus*). However, the Tilarán range in northern Costa Rica is estimated to have originated around 2 Myr BP [57], after the present split between Caribbean and Pacific lineages. Sister species are often found on opposite sides of the Central American Highlands [20, 21], and while Caribbean lineages of *N. humilis* form a paraphyletic group, the fact that one clade is separated by the continental divide is unsurprising. The ancestral area estimation suggests that early ancestors of some of the lineages were present on both sides of the continental divide in Costa Rica and Panama, corresponding to dates prior to the rise of the Central American Highlands.

As in many other taxa ([58] and sources within), LCA has served as a region of diversification for the *Norops humilis/quaggulus* complex (once again, distinct from the *N. humilis* species group). The northward distribution observed here is similar to that found in eleutherodactyline (genera *Craugastor* [19]; *Pristimantis* [59]) and hylid frogs (*Dendropsophus* [60]) that originated in South America and dispersed to Central America prior to the most recent completion of the Isthmus of Panama (3–4 Myr BP). This is also similar to findings in other groups of squamates [2], although it remains to be seen if this pattern of dispersal is shared by other lizard species.

Our third objective was to examine polyphyly described by others [35, 36] of the *N. humilis* species group. The lack of support for a monophyletic *N. humilis* species group is consistent with results from Nicholson [35] and Poe [36], further illustrating the extent of convergence in morphological characters of mainland anoles. All analyses agreed in assigning several species of the *N. humilis* species group into separate areas of the tree. *Norops marsupialis, N. tropidonotus*, and *N. uniformis* were placed in areas of the tree distant to a monophyletic *N. humilis/quaggulus* complex. Initially *N. marsupialis* was included in this study as a member of the *N. humilis/quaggulus* complex*.* Taylor [61] described *N. marsupialis* as a subspecies of *N. humilis* in 1956, and it was not elevated to specific status until 2015 [34]. All of our analyses (Bayesian and ML for each gene region and combined) found support for the specific status of *N. marsupialis*, and agreed to its placement as sister to a clade containing *N. aquaticus* and *N. woodi* (approx. 30 Myr divergent from the *N. humilis/quaggulus* complex, Fig. 6). Therefore, we confirm *N. marsupialis* as a distinct species morphologically similar to, but evolutionary distant from *N. humilis/quaggulus*, in concordance with with Köhler et al. [34]*.* Morphological convergence within anoles has long been reported, particularly for Caribbean species [27, 62–66] and is further demonstrated by our analysis of the *N. humilis* species group. Inclusion of the other three members of the group (*N. compressicauda, N. notopholis* and *N. wampuensis*) into the molecular phylogeny of *Norops* may yield further insight on their placement in the phylogeny, but their inclusion is not likely to significantly alter the results obtained here. Additional work within *N. tropidonotus* may also be necessary to examine the evolution that has occurred in that species, as it occupies a broad geographic range (Köhler 2008 [30]). Our data suggest that *N. tropidonotus* contains at least three deep mitochondrial divergences, and could potentially represent multiple cryptic lineages. While *N. quaggulus* is nested within *N. humilis*, we do not recommend synonymizing the two species until further work is done to investigate this complex. The deep divergences seen among the *N. humilis* clades may correspond to cryptic species, and if further work confirms the presence of two or more species, the name *N. quaggulus* would have priority as the senior synonym.

The SAMOVA results indicate that limited mitochondrial gene flow is occurring among localities, suggesting 12 distinct genetic groupings among our sample localities. Therefore, we conclude that genetic differentiation within the *N. humilis/quaggulus* complex is significant enough to conclude that (1) population fragmentation has occurred and (2) the complex does not represent a single panmictic population. The isolation, coupled with the high genetic variance, supports all major lineages identified in the Bayesian analyses as being distinct from one another, as well as further subdivision within most lineages. While we do not suggest that the 12 groups correspond to separate species, the presence of at least six well supported, divergent clades within the *N. humilis/quaggulus* complex demonstrates that deep mitochondrial divergence has occurred within this group, although nuclear evolution appears to be more conserved. This result is similar to several studies on Caribbean anoles, which also display considerable mitochondrial differentiation, with much less diversity in the nuclear DNA [67–70]. This pronounced phylogeographic substructuring may be explained in part, by low vagility in lizards [9]. Deep mitochondrial divergences coupled with conserved nuclear evolution as seen here, may have at least two implications 1) ITS is more slowly evolving than the mitochondrial genome of anoles, which we consider to be a likely scenario given that nuclear DNA is generally regarded as experiencing slower rates of evolution [71–73] and 2). The mitochondrial-nuclear relationships observed here are characteristic of male-biased dispersal [74, 75], indicating that female *N. humilis* are more philopatric than males as seen in Caribbean anoles [76, 77].

The novel biogeographic pattern for Central American anoles revealed here illustrates a need for further work on mainland *Norops*. What remains to be tested is whether the south-to-north dispersal route seen in the *N. humilis/quaggulus* complex is repeated in other *Norops* groups. In addition, it is important to clarify where the Central American *Norops* lineage originated, how it dispersed throughout the mainland, and when these events took place. Investigating the phylogeography of other widespread anoles may be highly informative towards understanding other distribution patterns of the Central American herpetofauna. There are several widespread *Norops* species and species complexes that vary in their ecological roles with corresponding morphological features. These are grouped into designations called ecomodes, which are distinct from ecomorphs, to accommodate mainland anoles (see [35]). Such an assortment of ecologically diverse anoles may provide good models for testing the biogeographic hypotheses discussed here. Further studies on mainland *Norops* species are needed to test if the cryptic diversity suggested here is present in other widespread species complexes within the genus.

## Additional files

Additional file 1: Appendix S1. Locality and sequence data for *Norops humilis* species group *sensu* Savage and Guyer [31]. ITS accession numbers are not applicable to all specimens, as many taxa were only sequenced for the mtDNA region. Additional file 2: Appendix S2. Genbank numbers for sequences used in this analysis from 65 *Norops* clade outgroups. mtDNA were from multiple studies [78–80]. All ITS sequences are from Nicholson et al. [80]. Not all taxa used had available ITS sequences. Additional file 3: Appendix S3. Populations used in the SAMOVA analysis. With a graphical representation of the F_CT_ and F_SC_ values for a range of genetic clusters as defined in the spatial analysis of molecular variance.
