# Supplementary material for: Biogeography and evolution of a widespread Central American lizard species complex: Norops humilis, (Squamata: Dactyloidae)
Source: BMC Evol Biol. 2015 Jul 19;15(1):143. doi: 10.1186/s12862-015-0391-4 (PMC4506609; doi:10.1186/s12862-015-0391-4)
Supplement: Additional file 1: Appendix S1. — Locality and sequence data for Norops humilis species group sensu Savage and Guyer [31]. ITS accession numbers are not applicable to all specimens, as many taxa were only sequenced for the mtDNA region. [file 12862_2015_391_MOESM1_ESM.pdf]

Species	Voucher	Country	Province	GPS	mtDNA	ITS
N. humilis 1	MCNH 1746	Panama	Bocas del Toro	N 9.30735 W 82.14266	KJ954114	n/a
N. humilis 1	MCNH 1747	Panama	Bocas del Toro	N 9.35381 W 82.25657	KJ954115	n/a
N. humilis 1	MCNH 1748	Panama	Bocas del Toro	N 9.30735 W 82.14266	KJ954116	n/a
N. humilis 1	USNM 53582	Panama	Bocas del Toro	N 9.24678 W 82.27668	KJ954036	n/a
N. humilis 1	USNM 53810	Panama	Bocas del Toro	N 9.21798 W 82.26604	KJ954106	n/a
N. humilis 1	USNM 54064	Panama	Bocas del Toro	N 9.21798 W 82.26604	KJ954107	n/a
N. humilis 1	USNM 54065	Panama	Bocas del Toro	N 9.21798 W 82.26604	KJ954108	KJ523027
N. humilis 1	USNM 59822	Panama	Bocas del Toro	N 9.13969 W 82.10914	KJ954109	n/a
N. humilis 1	USNM 59824	Panama	Bocas del Toro	N 9.13969 W 82.10915	KJ954035	n/a
N. humilis 1	JMS 213	Panama	Chiriquí	N 8.72190 W 82.23726	KJ954000	n/a
N. humilis 1	JMS 215	Panama	Chiriquí	N 8.72190 W 82.23726	KJ954001	n/a
N. humilis 1	JMS 217	Panama	Chiriquí	N 8.72190 W 82.23726	KJ954002	KJ523026
N. humilis 1	JMS 220	Panama	Chiriquí	N 8.72190 W 82.23726	KJ954003	n/a
N. humilis 1	JMS 223	Panama	Chiriquí	N 8.72190 W 82.23726	KJ954005	n/a
N. humilis 1	JMS 227	Panama	Chiriquí	N 8.72190 W 82.23726	KJ954006	n/a
N. humilis 1	MCNH 1743	Panama	Chiriquí	N 8.72190 W 82.23726	KJ954111	n/a
N. humilis 1	MCNH 1744	Panama	Chiriquí	N 8.72190 W 82.23726	KJ954112	n/a
N. humilis 1	MCNH 1745	Panama	Chiriquí	N 8.72190 W 82.23726	KJ954113	n/a
N. humilis 1	JMS 221	Panama	Chiriquí	N 8.72190 W 82.23726	KJ954004	n/a
N. humilis 2	38022	Costa Rica	Limon	N 9.73836 W 82.85549	KJ953924	KJ523016
N. humilis 2	DL 1370	Costa Rica	Limon	N 9.64259 W 82.79593	KJ953946	n/a
N. humilis 2	DL 1371	Costa Rica	Limon	N 9.64259 W 82.79593	KJ953947	n/a
N. humilis 2	DL 1372	Costa Rica	Limon	N 9.64259 W 82.79593	KJ953948	n/a
N. humilis 2	DL 1373	Costa Rica	Limon	N 9.64259 W 82.79593	KJ953949	n/a
N. humilis 2	DL 1374	Costa Rica	Limon	N 9.64259 W 82.79593	KJ953950	n/a
N. humilis 2	DL 1375	Costa Rica	Limon	N 9.64259 W 82.79593	KJ953951	KJ523017
N. humilis 2	DL 1376	Costa Rica	Limon	N 9.64259 W 82.79593	KJ953952	n/a
N. humilis 2	DL 1377	Costa Rica	Limon	N 9.64259 W 82.79593	KJ953953	n/a
N. humilis 2	DL 1378	Costa Rica	Limon	N 9.64259 W 82.79593	KJ953954	n/a
N. humilis 2	DL 1380	Costa Rica	Limon	N 9.64259 W 82.79593	KJ953955	n/a
N. humilis 2	DL 1381	Costa Rica	Limon	N 9.64259 W 82.79593	KJ953956	KJ523018
N. humilis 2	DL 1382	Costa Rica	Limon	N 9.64259 W 82.79593	KJ953957	n/a
N. humilis 2	DL 1383	Costa Rica	Limon	N 9.64259 W 82.79593	KJ953958	n/a
N. humilis 2	DL 1384	Costa Rica	Limon	N 9.64259 W 82.79593	KJ953959	n/a
N. humilis 2	DL 1385	Costa Rica	Limon	N 9.64259 W 82.79593	KJ953960	n/a
N. humilis 2	DL 1386	Costa Rica	Limon	N 9.64259 W 82.79593	KJ953961	n/a
N. humilis 2	DL 1389	Costa Rica	Limon	N 9.64259 W 82.79593	KJ953962	n/a
N. humilis 2	DL 1390	Costa Rica	Limon	N 9.64259 W 82.79593	KJ953963	n/a
N. humilis 2	DL 1391	Costa Rica	Limon	N 9.64259 W 82.79593	KJ953964	n/a
N. humilis 2	DL 1393	Costa Rica	Limon	N 9.64259 W 82.79593	KJ953965	n/a
N. humilis 2	DL 1394	Costa Rica	Limon	N 9.64259 W 82.79593	KJ953966	n/a
N. humilis 2	DL 1397	Costa Rica	Limon	N 9.64259 W 82.79593	KJ953967	n/a

N. humilis 2	DL 1398	Costa Rica	Limon	N 9.64259 W 82.79593	KJ953968	n/a
N. humilis 2	DL 1399	Costa Rica	Limon	N 9.64259 W 82.79593	KJ953969	n/a
N. humilis 2	DL 1400	Costa Rica	Limon	N 9.64259 W 82.79593	KJ953970	n/a
N. humilis 2	MCNH 1738	Costa Rica	Limon	N 9.61889 W82.66218	KJ954020	KJ523019
N. humilis 2	MCNH 1739	Costa Rica	Limon	N 9.61889 W82.66218	KJ954021	KJ523024
N. humilis 2	MCNH 1740	Costa Rica	Limon	N 9.61889 W82.66218	KJ954022	KJ523025
N. humilis 2	MCNH 1741	Costa Rica	Limon	N 9.61889 W82.66218	KJ954023	n/a
N. humilis 2	MCNH 1742	Costa Rica	Limon	N 9.61889 W82.66218	KJ954024	n/a
N. humilis 3	GK 1739	Costa Rica	Guanacaste	N 10.36392 W 84.80292	KJ954039	KJ523008
N. humilis 3	GK 1740	Costa Rica	Guanacaste	N 10.36392 W 84.80292	KJ954040	n/a
N. humilis 3	JMS 86	Costa Rica	Guanacaste	N 10.97982 W 85.47710.	KJ953994	n/a
N. humilis 3	JMS 90	Costa Rica	Guanacaste	N 10.97982 W 85.47710	KJ953995	KJ523009
N. humilis 3	JMS 93	Costa Rica	Guanacaste	N 10.97982 W 85.47710	KJ953996	n/a
N. humilis 3	JMS 96	Costa Rica	Guanacaste	N 10.97982 W 85.47710	KJ953997	n/a
N. humilis 3	JMS 97	Costa Rica	Guanacaste	N 10.97982 W 85.47710	KJ953998	n/a
N. humilis 3	JMS 98	Costa Rica	Guanacaste	N 10.97982 W 85.47710	KJ953999	n/a
N. humilis 3	MCNH 1735	Costa Rica	Guanacaste	N 10.90167 W 85.28859	KJ954017	n/a
N. humilis 3	MCNH 1736	Costa Rica	Guanacaste	N 10.90167 W 85.28859	KJ954018	KJ523010
N. humilis 3	38049	Costa Rica	Puntarenas	N 10.30826 W 84.80783	KJ953926	KJ523007
N. humilis 3	MCNH 1737	Costa Rica	Puntarenas	N 10.30826 W 84.80783	KJ954019	n/a
N. humilis 3	MONT 11	Costa Rica	Puntarenas	N 10.30826 W 84.80783	KJ954102	KJ523015
N. humilis 3	JS 961	Nicaragua	Rio San Juan	N 11.02270 W 85.05130	KJ954046	n/a
N. humilis 4	39568	Costa Rica	Limon	N 10.09380 W 83.38318	KJ953940	KJ523000
N. humilis 4	LLCC1	Costa Rica	Limon	N 10.09380 W 83.38318	KJ954031	KJ523001
N. humilis 4	LLCC13	Costa Rica	Limon	N 10.09380 W 83.38318	KJ954032	n/a
N. humilis 4	LLCC5	Costa Rica	Limon	N 10.09380 W 83.38318	KJ954033	KJ523002
N. humilis 5	MCNH 1725	Costa Rica	Cartago	N 9.87464 W 83.58406	KJ954007	n/a
N. humilis 5	MCNH 1726	Costa Rica	Cartago	N 9.88010 W 83.57035	KJ954008	KJ522992
N. humilis 5	MCNH 1727	Costa Rica	Cartago	N 9.87978 W 83.58476	KJ954009	KJ522991
N. humilis 5	MCNH 1728	Costa Rica	Cartago	N 9.87978 W 83.58476	KJ954010	KJ522998
N. humilis 5	MCNH 1729	Costa Rica	Cartago	N 9.87978 W 83.58476	KJ954011	n/a
N. humilis 5	MCNH 1730	Costa Rica	Cartago	N 9.92756 W 83.60104	KJ954012	n/a
N. humilis 5	MCNH 1731	Costa Rica	Cartago	N 9.92927 W 83.60046	KJ954013	n/a
N. humilis 5	MCNH 1732	Costa Rica	Cartago	N 9.92927 W 83.60046	KJ954014	n/a
N. humilis 5	MCNH 1733	Costa Rica	Cartago	N 9.87978 W 83.58476	KJ954015	n/a
N. humilis 5	MCNH 1734	Costa Rica	Cartago	N 9.98055 W 83.84563	KJ954016	KJ522999
N. humilis 5	38017	Costa Rica	Limon	N 10.03431 W 83.54179	KJ953944	KJ522988
N. humilis 5	39541	Costa Rica	Limon	N 10.09718 W 83.55003	KJ953928	KJ522989
N. humilis 5	39542	Costa Rica	Limon	N 10.09718 W 83.55003	KJ953929	n/a
N. humilis 5	39545	Costa Rica	Limon	N 10.09718 W 83.55003	KJ953931	KJ522990
N. humilis 5	39548	Costa Rica	Limon	N 10.09718 W 83.55003	KJ953932	n/a
N. humilis 5	39551	Costa Rica	Limon	N 10.09718 W 83.55003	KJ953934	n/a
N. humilis 5	39553	Costa Rica	Limon	N 10.09718 W 83.55003	KJ953935	n/a

N. humilis 5	39556	Costa Rica	Limon	N 10.09718 W 83.55003	KJ953937	n/a
N. humilis 5	39557	Costa Rica	Limon	N 10.09718 W 83.55003	KJ953938	n/a
N. humilis 5	39559	Costa Rica	Limon	N 10.09718 W 83.55003	KJ953939	n/a
N. humilis 5	39574	Costa Rica	Limon	N 10.03431 W 83.54179	KJ954110	n/a
N. humilis 5	GK 1936	Costa Rica	Limon	N 10.03431 W 83.54179	KJ954038	n/a
N. marsupialis	ALF 2	Costa Rica	San Jose	N 9.30861 W 83.77662	KJ953941	n/a
N. marsupialis	ALF 3	Costa Rica	San Jose	N 9.30861 W 83.77662	KJ953942	KJ523022
N. marsupialis	ALF JUV2	Costa Rica	San Jose	N 9.30861 W 83.77662	KJ953943	n/a
N. marsupialis	DL 1472	Costa Rica	San Jose	N 9.30861 W 83.77662	KJ953980	n/a
N. marsupialis	DL 1473	Costa Rica	San Jose	N 9.30861 W 83.77662	KJ953981	n/a
N. marsupialis	DL 1474	Costa Rica	San Jose	N 9.30590 W 83.77789	KJ953982	n/a
N. marsupialis	DL 1477	Costa Rica	San Jose	N 9.30590 W 83.77789	KJ953983	n/a
N. marsupialis	DL 1478	Costa Rica	San Jose	N 9.30590 W 83.77789	KJ953984	n/a
N. marsupialis	DL 1480	Costa Rica	San Jose	N 9.30590 W 83.77789	KJ953985	KJ523023
N. marsupialis	DL 1481	Costa Rica	San Jose	N 9.30861 W 83.77662	KJ953986	n/a
N. marsupialis	DL 1482	Costa Rica	San Jose	N 9.30861 W 83.77662	KJ953987	n/a
N. marsupialis	DL 1483	Costa Rica	San Jose	N 9.30861 W 83.77662	KJ953988	n/a
N. marsupialis	DL 1484	Costa Rica	San Jose	N 9.30861 W 83.77662	KJ953989	n/a
N. marsupialis	DL 1486	Costa Rica	San Jose	N 9.30861 W 83.77662	KJ953990	n/a
N. marsupialis	DL 1487	Costa Rica	San Jose	N 9.30861 W 83.77662	KJ953991	n/a
N. marsupialis	DL 1489	Costa Rica	San Jose	N 9.30861 W 83.77662	KJ953992	n/a
N. marsupialis	TINIM JUV2	Costa Rica	San Jose	N 9.30590 W 83.77789	KJ954103	n/a
N. quaggulus	DL 1352	Costa Rica	Alajuela	N 10.20960 W 84.16429	KJ953945	KJ522985
N. quaggulus	GK 2189	Costa Rica	Alajuela	N 10.68655 W 84.18048	KJ954042	n/a
N. quaggulus	DL 1404	Costa Rica	Heredia	N 10.43061 W 84.00347	KJ953971	n/a
N. quaggulus	DL 1405	Costa Rica	Heredia	N 10.43061 W 84.00347	KJ953972	n/a
N. quaggulus	DL 1406	Costa Rica	Heredia	N 10.43061 W 84.00347	KJ953973	KJ522986
N. quaggulus	DL 1407	Costa Rica	Heredia	N 10.43061 W 84.00347	KJ953974	n/a
N. quaggulus	DL 1408	Costa Rica	Heredia	N 10.43061 W 84.00347	KJ953975	n/a
N. quaggulus	DL 1409	Costa Rica	Heredia	N 10.43061 W 84.00347	KJ953976	n/a
N. quaggulus	DL 1417	Costa Rica	Heredia	N 10.43061 W 84.00347	KJ953977	n/a
N. quaggulus	DL 1418	Costa Rica	Heredia	N 10.43061 W 84.00347	KJ953978	n/a
N. quaggulus	DL 1419	Costa Rica	Heredia	N 10.43061 W 84.00347	KJ953979	KJ522987
N. quaggulus	LS RS 1	Costa Rica	Heredia	N 10.43061 W 84.00347	KJ954034	n/a
N. quaggulus	LS STR 20	Costa Rica	Heredia	N 10.43061 W 84.00347	KJ954100	n/a
N. quaggulus	LS STR 30	Costa Rica	Heredia	N 10.43061 W 84.00347	KJ954101	n/a
N. quaggulus	TIR 10	Costa Rica	Heredia	N 10.39956 W 84.14089	KJ954104	KJ523006
N. quaggulus	TIR CC 25	Costa Rica	Heredia	N 10.39956 W 84.14089	KJ954105	n/a
N. quaggulus	39544	Costa Rica	Limon	N 10.09718 W 83.55003	KJ953930	n/a
N. quaggulus	39550	Costa Rica	Limon	N 10.09718 W 83.55003	KJ953933	n/a
N. quaggulus	39555	Costa Rica	Limon	N 10.09718 W 83.55003	KJ953936	n/a
N. quaggulus	GK 1940	Costa Rica	Limon	N 10.26312 W 83.69775	KJ954041	KJ522993
N. quaggulus	MCNH 1769	Costa Rica	Limon	N 10.55862 W 83.73610	KJ954025	n/a

N. quaggulus	MCNH 1770	Costa Rica	Limon	N 10.55862 W 83.73610	KJ954026	KJ523004
N. quaggulus	MCNH 1771	Costa Rica	Limon	N 10.55862 W 83.73610	KJ954027	n/a
N. quaggulus	MCNH 1772	Costa Rica	Limon	N 10.55862 W 83.73610	KJ954028	n/a
N. quaggulus	MCNH 1773	Costa Rica	Limon	N 10.55862 W 83.73610	KJ954029	n/a
N. quaggulus	USNM 549361	Honduras	Gracias a Dios	N 14.94048 W 84.51296	KJ954073	n/a
N. quaggulus	USNM 549362	Honduras	Gracias a Dios	N 14.94048 W 84.51296	KJ954074	n/a
N. quaggulus	LDW 11739	Honduras	Olancho	N 14.81439 W 85.75716	KJ954030	KJ523005
N. quaggulus	H-349	Honduras	Olancho	N 14.81439 W 85.75716	KJ953993	n/a
N. quaggulus	N 911	Nicaragua	Jinotega	N 13.60429 W 85.68850	KJ954071	n/a
N. quaggulus	MCNH 1753	Nicaragua	Boaco	N 12.53579 W 85.35665	KJ954055	n/a
N. quaggulus	MCNH 1754	Nicaragua	Boaco	N 12.53437 W 85.36268	KJ954056	n/a
N. quaggulus	MCNH 1755	Nicaragua	Boaco	N 12.53437 W 85.36268	KJ954057	KJ522995
N. quaggulus	MCNH 1756	Nicaragua	Boaco	N 12.53437 W 85.36268	KJ954058	n/a
N. quaggulus	MCNH 1757	Nicaragua	Boaco	N 12.53437 W 85.36268	KJ954059	n/a
N. quaggulus	MCNH 1758	Nicaragua	Boaco	N 12.53437 W 85.36268	KJ954060	n/a
N. quaggulus	JHT 2281	Nicaragua	Jinotega	N 13.60429 W 85.68850	KJ954037	n/a
N. quaggulus	JS 1405	Nicaragua	Jinotega	N 13.15735 W 85.87385	KJ954048	n/a
N. quaggulus	JS 1674	Nicaragua	Jinotega	N 13.15735 W 85.87385	KJ954050	n/a
N. quaggulus	MCNH 1749	Nicaragua	Jinotega	N 13.15785 W 85.87355	KJ954051	n/a
N. quaggulus	MCNH 1750	Nicaragua	Jinotega	N 13.15785 W 85.87355	KJ954052	KJ522994
N. quaggulus	MCNH 1751	Nicaragua	Jinotega	N 13.15785 W 85.87355	KJ954053	n/a
N. quaggulus	MCNH 1752	Nicaragua	Jinotega	N 13.15785 W 85.87356	KJ954054	n/a
N. quaggulus	JS 1632	Nicaragua	Rio San Juan	N 10.93101 W 83.72604	KJ954049	n/a
N. quaggulus	JS 1634	Nicaragua	Rio San Juan	N 10.93101 W 83.72604	KJ954047	n/a
N. quaggulus	MCNH 1759	Nicaragua	Rio San Juan	N 10.97336 W 84.33887	KJ954061	n/a
N. quaggulus	MCNH 1760	Nicaragua	Rio San Juan	N 10.97336 W 84.33887	KJ954062	KJ522996
N. quaggulus	MCNH 1761	Nicaragua	Rio San Juan	N 10.97336 W 84.33887	KJ954063	n/a
N. quaggulus	MCNH 1762	Nicaragua	Rio San Juan	N 10.97336 W 84.33887	KJ954064	n/a
N. quaggulus	MCNH 1763	Nicaragua	Rio San Juan	N 10.97336 W 84.33887	KJ954065	n/a
N. quaggulus	MCNH 1764	Nicaragua	Rio San Juan	N 10.93101 W 83.72604	KJ954066	n/a
N. quaggulus	MCNH 1765	Nicaragua	Rio San Juan	N 10.93101 W 83.72604	KJ954067	KJ522997
N. quaggulus	MCNH 1766	Nicaragua	Rio San Juan	N 10.93101 W 83.72604	KJ954068	n/a
N. quaggulus	MCNH 1767	Nicaragua	Rio San Juan	N 10.84046 W 83.76687	KJ954069	n/a
N. quaggulus	MCNH 1768	Nicaragua	Rio San Juan	N 10.84046 W 83.76687	KJ954070	KJ523003
N. quaggulus	38045	Costa Rica	Heredia	N 10.21379 W 83.90114	KJ953925	KJ522983
N. quaggulus	39537	Costa Rica	Limon	N 9.48748 W 82.95240	KJ953927	KJ522984
N. tropidonotus	CAC 043	Honduras	Atlántida	N 15.67231 W 87.14298	KJ954077	n/a
N. tropidonotus	CAC 048	Honduras	Atlántida	N 15.73841 W 86.85047	KJ954078	n/a
N. tropidonotus	CAC 050	Honduras	Atlántida	N 15.73841 W 86.85047	KJ954079	n/a
N. tropidonotus	CAC 052	Honduras	Atlántida	N 15.73841 W 86.85047	KJ954080	KJ523012
N. tropidonotus	CAC 053	Honduras	Atlántida	N 15.73841 W 86.85047	KJ954081	n/a
N. tropidonotus	USNM 539839	Honduras	Atlántida	N 15.62999 W 86.76670	KJ954072	n/a
N. tropidonotus	JHT 2354	Honduras	Comayagua	N 14.50517 W 87.49317	KJ954087	n/a

N. tropidonotus	JHT 2961	Honduras	Comayagua	N 14.50517 W 87.49317	KJ954090	n/a
N. tropidonotus	JHT 1404	Honduras	Cortes	N 15.37815 W 87.95494	KJ954045	n/a
N. tropidonotus	JHT 2459	Honduras	El Paraiso	N 13.73906 W 86.95330	KJ954044	n/a
N. tropidonotus	JHT 2147	Honduras	Francisco Morazán	N 14.01787 W 87.08256	KJ954085	n/a
N. tropidonotus	JHT 2303	Honduras	Francisco Morazán	N 14.99421 W 87.09080	KJ954043	n/a
N. tropidonotus	JHT 2306	Honduras	Francisco Morazán	N 14.45364 W 87.05921	KJ954086	n/a
N. tropidonotus	JHT 2962	Honduras	Francisco Morazán	N 14.45364 W 87.05921	KJ954091	n/a
N. tropidonotus	CAC 006	Honduras	Intibucá	N 14.30698 W 88.17707	KJ954076	KJ523011
N. tropidonotus	CAC 005	Honduras	Olancho	N 14.49553 W 86.51247	KJ954075	n/a
N. tropidonotus	JHT 2424	Honduras	Santa Bárbara	N 15.12393 W 88.40263	KJ954088	n/a
N. tropidonotus	JHT 3006	Honduras	Santa Bárbara	N 15.12393 W 88.40263	KJ954092	KJ523020
N. tropidonotus	JHT 2127	Honduras	Yoro	N 15.20735 W 87.60029	KJ954084	n/a
N. tropidonotus	JHT 2577	Honduras	Yoro	N 15.30672 W 87.12513	KJ954089	KJ523014
N. tropidonotus	CSL 6359	Mexico	Veracruz	N 18.85108 W 97.06997	KJ954083	KJ523013
N. tropidonotus	CSL 6368	Mexico	Veracruz	N 18.85108 W 97.06997	KJ954082	n/a
N. uniformis	USNM 1299	Belize	Toledo	N. 16.27128 W 88.94892	KJ954093	n/a
N. uniformis	USNM 1306	Belize	Toledo	N. 16.34773 W 88.98119	KJ954094	n/a
N. uniformis	USNM 1307	Belize	Toledo	N. 16.34773 W 88.98119	KJ954095	n/a
N. uniformis	USNM 1309	Belize	Toledo	N. 16.34773 W 88.98119	KJ954096	KJ523021
N. uniformis	USNM 1311	Belize	Toledo	N. 16.34773 W 88.98119	KJ954097	n/a
N. uniformis	USNM 1312	Belize	Toledo	N. 16.34773 W 88.98119	KJ954098	n/a
N. uniformis	USNM 1313	Belize	Toledo	N. 16.34773 W 88.98119	KJ954099	n/a
