# Supplementary material for: Biogeography and evolution of a widespread Central American lizard species complex: Norops humilis, (Squamata: Dactyloidae)
Source: BMC Evol Biol. 2015 Jul 19;15(1):143. doi: 10.1186/s12862-015-0391-4 (PMC4506609; doi:10.1186/s12862-015-0391-4)
Supplement: Additional file 2: Appendix S2. — Genbank numbers for sequences used in this analysis from 65 Norops clade outgroups. mtDNA were from multiple studies [78–80]. All ITS sequences are from Nicholson et al. [80]. Not all taxa used had available ITS sequences. [file 12862_2015_391_MOESM2_ESM.pdf]

Species	Mitochondrial	ITS	Source for mtDNA
N. ahli	AY296148	KF728963	Harmon *et al*. 2003
N. alayoni	AY296149	n/a	Harmon *et al*. 2003
N. allogus	AY296152	KF728964	Harmon *et al*. 2003
N. altae	AY909735	KF728965	Nicholson *et al*. 2005
N. annectens	AY909736	n/a	Nicholson *et al*. 2005
N. aquaticus	AY909738	KF728966	Nicholson *et al*. 2005
N. auratus	AY909740	KF728967	Nicholson *et al*. 2005
N. bicaorum	AY909741	n/a	Nicholson *et al*. 2005
N. biporcatus	AF294286	KF728968	Jackman *et al*. 2002
N. bitectus	AY909743	KF728969	Nicholson *et al*. 2005
N. bremeri	AY296157	KF728970	Harmon *et al*. 2003
N. capito	AY909744	KF728971	Nicholson *et al*. 2005
N. carpenteri	AY296160	KF728972	Harmon *et al*. 2003
N. confusus	AY909787	n/a	Nicholson *et al*. 2005
N. conspersus	AF294304	n/a	Jackman *et al*. 2002
N. crassulus	AY909748	KF728974	Nicholson *et al*. 2005
N. cupreus	AY909750	KF728975	Nicholson *et al*. 2005
N. fuscoauratus	AF337792	KF728977	Genbank Direct Submission
N. garmani	AF294289	KF728978	Jackman *et al*. 2002
N. grahami	AF294303	KF728980	Jackman *et al*. 2002
N. guafe	AY909788	n/a	Nicholson *et al*. 2005
N. homolechis	AY296179	KF728981	Harmon *et al*. 2003
N. humilis	AF055944	KF728982	Jackman *et al*. 2002
N. intermedius	AY909755	KF728983	Nicholson *et al*. 2005
N. isthmicus	AY909762	KF728984	Nicholson *et al*. 2005
N. jubar	AY296182	KF729017	Harmon *et al*. 2003
N. laeviventris	AY909756	KF728985	Nicholson *et al*. 2005
N. lemurinus	AF294283	KF728986	Jackman *et al*. 2002
N. limifrons	AF055943	KF729016	Jackman *et al*. 2002
N. lineatopus	AF294297	KF728987	Jackman *et al*. 2002
N. lionotus	AY909757	KF728988	Nicholson *et al*. 2005
N. loveridgei	AY909759	n/a	Nicholson *et al*. 2005
N. medemi	KJ953921	n/a	Nicholson *et al*. 2012
N. meridionalis	AY909760	n/a	Nicholson *et al*. 2005
N. mestrei	AF337779	KF728989	Genbank Direct Submission
N. nebuloides	AY909763	KF728990	Nicholson *et al*. 2005
N. nitens	AF337800	KF728991	Genbank Direct Submission
N. ocelloscapularis	AY909767	KF728992	Nicholson *et al*. 2005
N. onca	AY909765	n/a	Nicholson *et al*. 2005
N. opalinus	AF294305	n/a	Jackman *et al*. 2002
N. ophiolepis	AF294317	KF728993	Jackman *et al*. 2002

N. ortonii	AF294288	KF728994	Jackman *et al*. 2002
N. oxylophus	AY909768	KF728995	Nicholson *et al*. 2005
N. pachypus	AY909769	KF728996	Nicholson *et al*. 2005
N. pandoensis	AY909770	n/a	Nicholson *et al*. 2005
N. poecilopus	AY909771	KF728997	Nicholson *et al*. 2005
N. polylepis	AY909772	KF728998	Nicholson *et al*. 2005
N. polyrhachis	AY909773	n/a	Nicholson *et al*. 2005
N. purpurgularis	AY909774	n/a	Nicholson *et al*. 2005
N. quadriocellifer	AY296197	KF728999	Harmon *et al*. 2003
N. quercorum	AY909775	KF729000	Nicholson *et al*. 2005
N. reconditus	AY296198	KF729001	Harmon *et al*. 2003
N. sagrei	AF055940	KF729003	Jackman *et al*. 2002
N. sericeus	AY909778	KF729004	Nicholson *et al*. 2005
N. sminthus	AY909779	KF729005	Nicholson *et al*. 2005
N. newspecies	KJ953923	n/a	Nicholson *et al*. 2012
N. townsendi	KJ953922	n/a	Nicholson *et al*. 2012
N. trachyderma	AF294285	KF729006	Jackman *et al*. 2002
N. tropidogaster	AY909782	KF729007	Nicholson *et al*. 2005
N. tropidonotus	AY909783	KF729009	Nicholson *et al*. 2005
N. uniformis	AY909784	KF729010	Nicholson *et al*. 2005
N. utilensis	AY909785	n/a	Nicholson *et al*. 2005
N. valencienni	AF294310	KF729011	Jackman *et al*. 2002
N. woodi	AF337780	KF729012	Genbank Direct Submission
N. zeus	AY909786	n/a	Nicholson *et al*. 2005
