# Supplementary figures and images for: Biogeography and evolution of a widespread Central American lizard species complex: Norops humilis, (Squamata: Dactyloidae)
Source: BMC Evol Biol. 2015 Jul 19;15(1):143. doi: 10.1186/s12862-015-0391-4 (PMC4506609; doi:10.1186/s12862-015-0391-4)

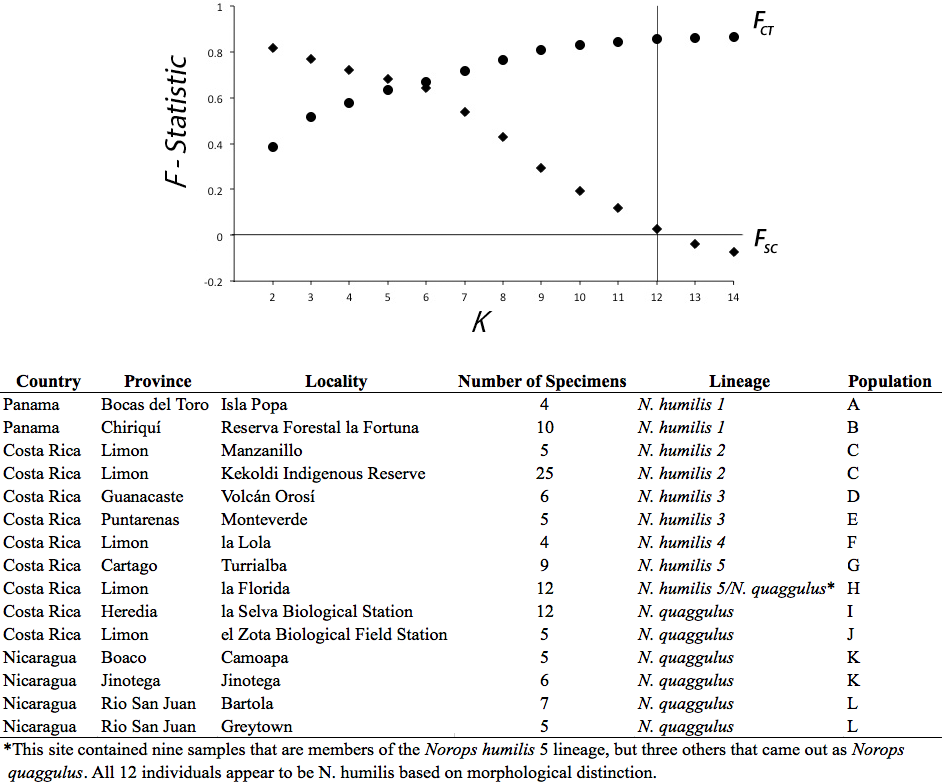

Supplement: Additional file 3: Appendix S3. — Populations used in the SAMOVA analysis. With a graphical representation of the FCT and FSC values for a range of genetic clusters as defined in the spatial analysis of molecular variance. [file 12862_2015_391_MOESM3_ESM.png]
